# Assessing the role of venetoclax in combination with hypomethylating agents in higher risk myelodysplastic syndrome

**DOI:** 10.1038/s41408-022-00744-z

**Published:** 2022-11-04

**Authors:** Rami S. Komrokji, Avani M. Singh, Najla Al Ali, Onyee Chan, Eric Padron, Kendra Sweet, Andrew Kuykendall, Jeffrey E. Lancet, David A. Sallman

**Affiliations:** 1 Department of Malignant Hematology, H. Lee Moffitt Cancer Center & Research Institute, Tampa, FL USA; 2 Department of Hematology/Oncology, University of South Florida & Moffitt Cancer Center, Tampa, FL USA

**Keywords:** Cancer therapy, Myelodysplastic syndrome

Dear Editor,

Myelodysplastic syndromes (MDS) are a myeloid neoplasm characterized by clonal hematopoiesis, peripheral cytopenias, and morphologic dysplasia with a tendency to progress to acute myeloid leukemia. Current therapies are tailored based on disease risk using the Revised International Prognostic System (IPSS-R) complemented by molecular data and patient-related factors [1, 2]. The standards of care for high risk MDS (HR-MDS) are hypomethylating agents (HMA) and allogeneic hematopoietic stem cell transplantation (AHSCT), which remains the only curative option. HR-MDS overall response rates to HMA therapy approach 50%, although <20% achieve complete response (CR) [3]. The original AZA-001 MDS studies demonstrated a median OS of 24 months, while several real-world data have reported a median OS of only 15–17 months [4, 5]. *TP53* and *ASXL1* somatic mutations have been associated with worse OS and inferior response to HMA [6]. After HMA failure, outcomes are poor with median OS being 4–6 months and treatment options are limited [7–9]. Venetoclax is a BH3 mimetic which binds to BCL-2, an antiapoptotic protein, ultimately inducing cellular apoptosis [3]. The combination of HMA and Venetoclax has become the standard of care treating AML patients unfit for intensive chemotherapy where randomized clinical trials have demonstrated OS advantage for the combination. We compared outcomes of HR-MDS patients who have been treated with HMAs alone and HMA with Venetoclax, in combination as first line (1 L) therapy and in sequence as an add-back strategy at time of HMA failure.

HR-MDS patients, defined by IPSS-R by ≥ intermediate risk, treated at Moffitt Cancer Center who received an HMA as 1 L therapy after diagnosis were included in this analysis. We compared outcomes of those patients who received single agent 1 L HMA, 1 L HMA/Venetoclax combination, and HMA with Venetoclax add-back strategy after HMA failure later. The decision to treat with combination therapy was based on physician choice. HMA failure was defined as progression on therapy or lack of response after at least 4 cycles. Response to treatment was based on the International Working Group 2006 criteria. Overall survival was defined from time of diagnosis.

We identified 1193 HR-MDS patients who received HMA as 1 L therapy. 1158 patients received 1 L single agent HMA (1027 received azacitidine and 131 received decitabine), and 35 patients received 1 L HMA/Venetoclax combination (26 received azacitidine and 9 received decitabine). Among the 1158 patients with 1 L HMA alone, 31 were subsequently treated with HMA/Venetoclax combination at the time of HMA failure without transformation to AML. The median duration of follow up from diagnosis was 96 months for 1 L HMA, 15 months for 1 L HMA/Ven, and 36mo for HMA/Ven in relapsed/refractory (R/R) MDS. Table 1A summarizes baseline clinical characteristics. For 1 L treatment, patients who received 1 L HMA/Venetoclax were more likely to be categorized as MDS-EB2 based on WHO 2016 classification and more likely to harbor the *ASXL1* somatic mutation. The median time to initiate treatment from time of diagnosis was 1 month with no difference between the two arms. Venetoclax starting dose was 400 mg PO daily on days 1–14 of each 28-day cycle. The ven dose was adjusted as needed for antibiotic prophylaxis per package insert guidelines. The median number of treatment cycles administered was 5 for HMA alone and 4 cycles for HMA/Ven.

**Table 1:** A Baseline patient characteristics. B Best response rates to first-line therapy.

		HMA IL	HMA/Ven 1 L	*P-*value	HMA/VEN R/R cohort
**A**					
**N**		1127	35		31
**Age (years)**	mean	68.4	67.8	0.76	70.9
**Gender**	Male	66%	71%	0.50	74%
**Race**	White	90%	97%	0.66	94%
**t-MDS**		24%	23%	0.86	26%
**WHO 2016**	MDS-SLD/MLD	18%	4%	0.04	19%
	MDS-RS	6%	4%		3%
	MDS-EB1	33%	9%		36%
	**MDS-EB2**	39%	78%		42%
**IPSS-R**	Intermediate	31%	17%	0.22	45%
	High	31%	37%		32%
	Very High	38%	46%		23%
**Myeloblasts (%)**	Mean	8	13	<0.005	9%
**Hgb (g/dL)**	Mean	9	9	1.00	9.9
**WBC (k/uL)**	Mean	4	10.6	<0.005	3.1
**ANC (k/uL)**	Mean	1.8	4.1	<0.005	1.5
**Platelets (k/uL)**	mean	96	100	0.80	120
**Somatic Mutations (*n*** = 546 sequenced)	*SF3B1*	5%	0	0.30	10%
	*TET-2*	16%	23%	0.30	23%
	*IDH-1*	3%	3%	0.70	6%
	*IDH-2*	5%	14%	0.056	0
	***ASXL-1***	21%	46%	0.002	42%
	*TP53*	27%	34%	0.60	23%
	*NRAS*	4%	11%	0.07	3%
**B**					
	**HMA IL**	**HMA/Ven 1** **L**	***P*-value**		
**N**	1127	35			
**ORR**	40%	77%	<0.005		
**CR**	13%	34%			
**mCR**	11%	37% (62%+ HI)			
**PR**	1%	3%			
**HI**	15%	3%			
***ASXL1*** **MT**	*N* = 106	*N* = 16			
**ORR**	32%	87%	<0.005		
**CR**	8%	44%			
***TP53*** **MT**	*N* = 137	*N* = 12			
**ORR**	44%	75%	0.038		
**CR**	17%	25%	0.47		

The OS was 21 months (95% CI 11–32) and 20 months (95% CI 19–22) for 1 L HMA/Venetoclax and 1 L HMA alone respectively (*p* = 0.86) (Fig. 1A). Among the 269 patients who proceeded to allogeneic stem cell transplantation (AHSCT), 13 patients received 1 L HMA/Venetoclax. For these patients, the median OS was not reached compared to 38 months among those who received 1 L HMA alone (*p* = 0.20) (Fig. 1B). For patients who proceeded to AHSCT, the 2-year survival probability rates were 91% and 51% for 1 L HMA/Venetoclax and HMA alone, respectively. The rates of AML transformation were 23% and 37% for 1 L HMA/Venetoclax and HMA alone respectively (*p* = 0.08).

**Fig. 1:**
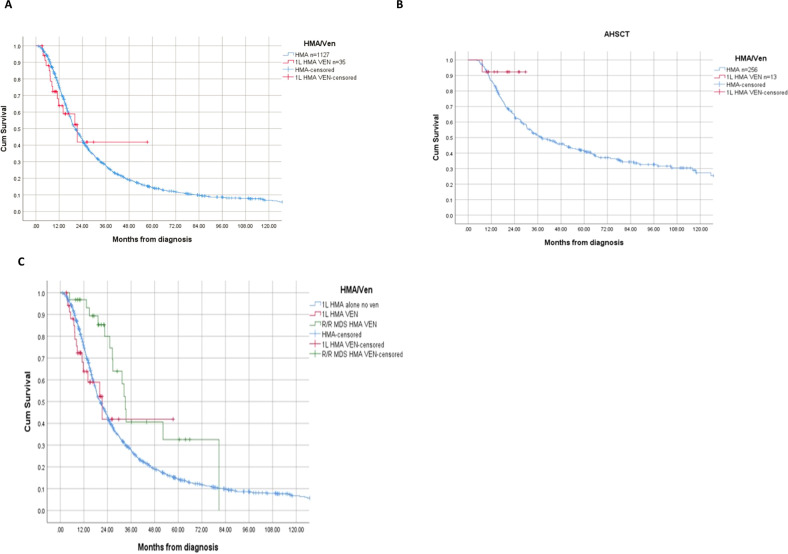
Kaplan Meier estimates of overall survival. **A** Overall survival in first-line HMA vs. HMA/Ven. **B** Overall survival among AHSCT cohort based on upfront therapy. **C** Overall survival in HMA/Ven in relapsed/refractory MDS after first-line HMA.

The ORR, defined as hematological improvement or better, was 77% for 1 L HMA/Venetoclax compared to 40% for 1 L HMA alone (*p* < 0.005). The complete response (CR)/ marrow CR (mCR)/ partial response (PR)/ hematologic improvement (HI) were 34%/37%/3%/3% compared to 13%/11%/1%/15% for 1 L HMA/Venetoclax and 1 L HMA alone respectively (*p* < 0.005). Among patients with *ASXL1* somatic mutation, the ORR was 87% and 32% for HMA/Venetoclax and HMA alone, respectively (*p* < 0.005). CR were 44% and 8% for HMA/Venetoclax and HMA alone respectively among *ASXL-1* mutant cohort (*p* < 0.005). Among patients with *TP53* somatic mutation, the ORR was 75% and 44% for 1 L HMA/Venetoclax and HMA alone respectively (*p* = 0.038), however CR were 25% and 17% respectively (*p* = 0.47) (Table 1B).

Among patients who received 1 L HMA alone, 31 patients later received HMA/Venetoclax for R/R MDS. The median number of prior 1 L HMA therapy alone was 6 cycles. For this cohort, the ORR was 61%, CR 13%, and mCR 48%. The median OS from diagnosis for patients who received HMA/Venetoclax for HMA failure MDS was 33 months (95% CI 31–36). This is compared to 21 months (95% CI 11–32) for those who had 1 L HMA/Venetoclax and 20 months (95% CI 19–22) for those who received 1 L HMA alone with no subsequent Venetoclax therapy (*p* = 0.02) (Fig. 1C). Nine of 31 patients who received HMA/Venetoclax for R/R MDS underwent AHSCT compared to 22 who did not proceed to transplant with median OS of 31 months and 33 months, respectively (*p* = 0.70).

In this large retrospective study among HR-MDS patients, 1 L HMA/Venetoclax combination therapy resulted in significantly higher CR rates compared to 1 L HMA alone. This data confirms the higher response of combination HMA/Venetoclax observed in recent phase I clinical trials and retrospective reviews. In a phase 1b trial, treatment-naïve HR-MDS patients were treated with Aza/Ven. Results demonstrated mORR of 80%, including CR of 40%, and mCR of 40% [10]. OS rate at 12 months was 94% for patients who reached a CR and 74% for those who reached a mCR.

An ongoing phase 1b, open-label, multicenter study conducted by Zeidan et al. evaluates the safety of Venetoclax and Azacitidine for 44 patients with R/R MDS. Results revealed an ORR of 39% with 32% mCR and 7% CR [7]. Of those with mCR, 86% had platelet transfusion independence (TI), 71% had red blood cell TI, and 6% had complete TI. Median OS was 12.3 months for all patients and 14.8 months for those who reached mCR [7].

Forty patients with MDS-EB2 treated with HMA/Venetoclax at the Mayo Clinic were included in a retrospective analysis by Gangat et al. Sixteen patients were treatment-naïve, eight were HMA-exposed, and 16 were HMA-refractory. All IPSS-R risk categories were included, and 23 patients received decitabine and 17 patients received azacitadine. Thirty-eight patients were included in final analyses, and results demonstrated 30% with CR, 37.5% with mCR, and of those with mCR, 27% had HI [11]. Of the 27 patients with CR or mCR, 11 patients proceeded to AHSCT. No difference in CR/mCR rates were found between those who were HMA-exposed, HMA-failure, or HMA naïve. HMA/Venetoclax CR/mCR was more likely in patients with *ASXL1*, *SRSF2*, or *IDH2* [11].

The OS benefit for 1 L HMA/Venetoclax in HR-MDS patients can only be addressed in context of a randomized clinical trial. We did not observe OS benefit for the whole cohort, though limited by small number of patients treated with the combination and short duration of follow up. Our data suggest promising activity amongst those who received 1 L HMA/Venetoclax and proceeded to AHSCT, with a 2-year OS of 91% compared to a 2-year OS of 51% with 1 L HMA alone. Based on our study and data from clinical trials, Venetoclax add-back strategy after 1 L HMA failure has clinical activity, including OS benefit compared to historical data and disease control to pursue AHSCT. We assessed OS from time of diagnosis, and those who received Venetoclax add-back strategy had the longest OS. This can be attributed partially to time lead bias where those patients had a response or stable disease with original HMA treatment alone, but these results also pose the question of the optimal timing and sequence of using Venetoclax in MDS.

Our data highlights improved ORR for patients with the poor-prognosis associated mutations of *ASXL1* and *TP53* treated with 1 L HMA/Venetoclax. The CR for 1 L HMA/Venetoclax vs. 1 L HMA in *ASXL1* patients was additionally significant at 44% compared to 8% respectively (*p* ≤ 0.005). Our clinical observation of higher responses with combination therapy among *ASXL1* mutant MDS patients supports the recent preclinical data suggesting that *ASXL1* resistance to azacitidine is mediated by overexpression of BCL-2, and addition of Venetoclax may overcome this resistance mechanism [12]. No difference in response was observed based on other mutations, however sample size of those subsets will preclude meaningful analysis.

Limitations of our study include its retrospective nature, small number of patients treated with HMA/Venetoclax combination, short duration of follow up for the combination arm, lack of minimal residual disease assessment, and the physician’s bias in administering the combination for selected group of patients. No data on dose adjustment and adverse events were collected retrospectively for this study purpose. Lastly, we acknowledge the discrepancy between the percentage of patients in each group that proceeded to AHSCT.

In conclusion, the historical control arm with HMA alone is one of the largest reported and both response rates and survival are consistent with previous reports. The response rates with combination therapy are encouraging, particularly among patients with known low response rates to HMA alone. The 2-year survival probability after AHSCT in this study is encouraging, as well as the activity noted after HMA failure. Randomized clinical trials are required to adequately understand the effectiveness of combination therapy of HMAs and Venetoclax as well as treatment with Venetoclax add-back strategy to change our current standard of care.

## Data Availability

The datasets generated during and/or analyzed during the current study are available from the corresponding author on reasonable request.

## References

[1] Ball BJ, Famulare CA, Stein EM, Tallman MS, Derkach A, Roshal M (2020). Venetoclax and hypomethylating agents (HMAs) induce high response rates in MDS, including patients after HMA therapy failure. Blood Adv.

[2] Nazha A, Komrokji R, Meggendorfer M, Jia X, Radakovich N, Shreve J (2021). Personalized prediction model to risk stratify patients with myelodysplastic syndromes. J Clin Oncol.

[3] Saygin C, Carraway HE (2021). Current and emerging strategies for management of myelodysplastic syndromes. Blood Rev.

[4] Zeidan AM, Sekeres MA, Garcia-Manero G, Steensma DP, Zell K, Barnard J (2016). Comparison of risk stratification tools in predicting outcomes of patients with higher-risk myelodysplastic syndromes treated with azanucleosides. Leukemia.

[5] Davidoff AJ, Hu X, Bewersdorf JP, Wang R, Podoltsev NA, Huntington SF (2020). Hypomethylating agent (HMA) therapy use and survival in older adults with Refractory Anemia with Excess Blasts (RAEB) in the United States (USA): a large propensity score-matched population-based study. (dagger) Leuk Lymphoma.

[6] Bejar R, Stevenson K, Abdel-Wahab O, Galili N, Nilsson B, Garcia-Manero G (2011). Clinical effect of point mutations in myelodysplastic syndromes. N. Engl J Med.

[7] Zeidan A, Pollyea, DA, Garcia, JS, Brunner AM, Roncolato F, Borate U, et al. 3109 A phase 1b study evaluating the safety and efficacy of venetoclax in combination with azacitadine for the treatment of relapsed/refractory myelodysplastic syndrome. Am Soc Hematol. 2020. https://ash.confex.com/ash/2020/webprogram/Paper136413.html.

[8] Prebet T, Gore SD, Esterni B, Gardin C, Itzykson R, Thepot S (2011). Outcome of high-risk myelodysplastic syndrome after azacitidine treatment failure. Research Support, Non-U.S. Gov’t. J Clin Oncol.

[9] Jabbour EJ, Garcia-Manero G, Strati P, Mishra A, Al Ali NH, Padron E (2015). Outcome of patients with low-risk and intermediate-1-risk myelodysplastic syndrome after hypomethylating agent failure: a report on behalf of the MDS Clinical Research Consortium. Cancer.

[10] Garcia JS, Wei AH, Jacoby MA, Fong CY, Borate U, Baer MR, et al. Molecular responses are observed across mutational spectrum in treatment-naive higher-risk myelodysplastic syndrome patients treated with venetoclax plus azacitadine. Am Soc Hematol. 2021. https://doi.org/10.1182/blood-2021-145613.

[11] Gangat N, McCullough K, Johnson I, Al-Kali A, Begna KH, Patnaik MM (2022). Real-world experience with venetoclax and hypomethylating agents in myelodysplastic syndromes with excess blasts. Am J Hematol.

[12] Rahmani NE, Ramachandra N, Sahu S, Gitego N, Lopez A, Pradhan K (2021). ASXL1 mutations are associated with distinct epigenomic alterations that lead to sensitivity to venetoclax and azacytidine. Blood Cancer J.

